# Literaturreview zu partizipativer Technologieentwicklung in der Behinderten- und Erziehungshilfe im Kontext Sozialer Arbeit

**DOI:** 10.1007/s12592-022-00439-z

**Published:** 2022-12-06

**Authors:** Tabea Mildenberger, Alena Schmier, Isabel Zorn

**Affiliations:** 1 TH Köln, Campus Südstadt, Sachsenring 2–4, 50677 Köln, Deutschland; 2 TH Köln, Campus Südstadt, Ubierring 48, 50678 Köln, Deutschland

**Keywords:** Eingliederungshilfe, Assistenztechnologie, Digitalisierung, Medienkompetenz, Partizipative Forschung, Social inclusion assistance, Service for disabled people, Assistive technology, Digitalization, Vulnerable youth

## Abstract

Ziel der Literaturstudie war es herauszufinden, welche wissenschaftlichen Projekte zu partizipativer Technikentwicklung in der Erziehungshilfe und jugendbezogener Behindertenhilfe bereits durchgeführt wurden und wie im wissenschaftlichen Diskurs der Sozialen Arbeit die Thematik partizipativer Technologieentwicklung in Einrichtungen der Jugend- und Behindertenhilfe behandelt wird. Ziel ist auch, die angewendete systematische Recherchestrategie zu beschreiben. Methodisch folgt der systematische Literaturreview einem definierten Korpus der relevanten, überwiegend deutschsprachigen wissenschaftlichen Fachzeitschriften Sozialer Arbeit zwischen 1994 und Mai 2020. Durchsucht wurden jene Fachzeitschriften, die sich mit Themengebieten angrenzend zur Forschungsfrage befassen: die Thematik der Jugend(hilfe), Medien, Technologie oder Digitalisierung. Der systematische Literaturreview benennt beforschte Felder und Themen. Die Ergebnisdarstellung erfolgt dabei kategorienbasiert entlang den Forschungslinien Partizipative Forschung, Assistenztechnologien, Handlungsanweisungen für Medienkompetenz von Jugendlichen und Digitalisierung in der Sozialen Arbeit. Der Beitrag zeigt Desiderata für zukünftige Forschung zu partizipativer Technikentwicklung in der Sozialen Arbeit auf.

## Abstract (en)

The aim of the literature study was to find out how the topic of participatory technology development in institutions for youth and disabled persons is treated in the scientific discourse of social work. A systematic literature review in a defined selection of relevant, predominantly German-language scientific journals on social work between 1994 and May 2020 was performed. Journals were searched for the following topics: youth (welfare), media, technology, or digitalization. The systematic literature review identified fields and topics of research. The results are presented based on categories according to the researched topics, i.e., participatory research, assistance technologies, guidance for media competence of young people, and digitalization in social work. The article points out potential areas for future research regarding participatory technology development in social work.

## Einleitung und Fragestellung

Der Stand der Digitalisierung in der jugendbezogenen Behinderten- und Erziehungshilfe ist nur ausschnittweise erforscht. Spätestens durch die COVID-19-Pandemie ist in der Praxis deutlich geworden, dass Handlungsbedarf besteht – sowohl in der Ausstattung als auch in den Nutzungskonzepten (Feyer et al. 2020). Es gilt bei der Ausstattung von Einrichtungen mitzudenken, welche Hilfsangebote digital unterstützt werden können oder sollten. Ebenso wie für die Durchschnittsbevölkerung könnten für Klient*innen der Sozialen Arbeit digitale Technologien förderlich für die Lebensbewältigung sein (Rocheleau et al. 2018; Dirks et al. 2018). Für gleichberechtigte digitale Teilhabe sind die benötigten Ausstattungen und Bildungsangebote vorzuhalten. Planungs- und Entwicklungsprozesse für die Auswahl und Implementierung neuer Technologien in stationären jugendbezogenen Wohnformen der Erziehungs- und Eingliederungshilfe könnten – entsprechend des Gebots der Partizipation an Unterstützungsmaßnahmen (SGB VIII) – partizipativ durchgeführt werden. Die Teilhabe an den Planungs- und Entwicklungsprozessen verspricht, sowohl für die Qualität der entwickelten Technologien als auch für die Ermächtigung und Bildung der beteiligten jungen Menschen zuträglich zu sein. Denn digitale Technologien stellen für die Jugendlichen mögliche Informations- und Kommunikationskanäle zu Bezugspersonen außerhalb von stationären Wohnformen dar. Diese Kanäle implizieren eine Erweiterung der Orientierungs- und Handlungsspielräume, sodass die Bewohner*innen der sozialen Einrichtungen einen Autonomiegewinn erfahren (Tillmann und Weßel 2021). Partizipative Entwicklungsprozesse ermächtigen die Jugendlichen zu einem informierten Umgang mit Technologien. Partizipation kann demnach förderlich für die Kompetenz- und Wissensvermittlung sein (Vaughn und Jacquez 2020). Die Klient*innen werden durch partizipative Prozesse zum Sprachrohr ihrer eigenen Bedürfnisse und somit im gesellschaftlichen Diskurs sichtbar (Straßburger und Rieger 2019).

Literaturrecherchen zur partizipativen Technikentwicklung in der Sozialen Arbeit weisen auf interdisziplinäre Forschungsprojekte mit Informatik oder Design hin. Die Publikationen thematisieren vorrangig Partizipation mit behinderten Menschen (Pareto et al. 2015; Guffroy et al. 2018; Heumader et al. 2018) oder mit Hochaltrigen sowie Menschen mit Demenzerkrankungen (Span et al. 2013) im Kontext der Gesundheitshilfe (Hamzah und Wahid 2016; Orlowski et al. 2015). Partizipative Ansätze in der Technikentwicklung mit Kindern sind zwar untersucht (Druin 2002; Nesset und Large 2004; Guha et al. 2005; Kafai et al. 2011), teilweise auch mit Kindern und Jugendlichen mit Behinderung (Benton und Johnson 2015; Börjesson et al. 2015; Weightman et al. 2010; Flores et al. 2018; Louw 2018), aber Ansätze innerhalb von (stationären) Eingliederungs- und Erziehungshilfen sind kaum vorhanden. Jugendliche in stationären Wohnformen zeigen jedoch einen besonderen Bedarf an partizipativer Technologienentwicklung auf: Wegen der Potenziale von Technologien für die Aufgabenerfüllung dieser Handlungsfelder in den Einrichtungen der Sozialen Arbeit (z. B. Planung von Hilfen – sowie (digitalen) Unterstützung bei der selbstbestimmten Lebensführung und der Verselbstständigung, Unterstützung im Kontext des ICF-Bereichs Aktivitäten und Partizipation), wegen der bislang geringen Vorschläge und Nutzungen von Technologien in stationären Wohnformen und wegen des Gebots der Partizipation der Klient*innen an der Planung von Unterstützungsmaßnahmen scheint es vielversprechend, die Potenziale partizipativer Technologieentwicklung durch Klient*innen und Fachkräfte in diesem Kontext zu erforschen. Nicht zuletzt die durch die Corona-Pandemiemaßnahmen sichtbar gewordenen Teilhabeproblematiken stationärer Einrichtungen bestärken den Handlungsbedarf in Digitalisierung und Partizipation der Jugendlichen in Technikentwicklungen. Digitalisierungsprozesse oder Technologien, die ohne Erprobungen unter den spezifischen Bedingungen (stationärer) Hilfekontexte der Sozialen Arbeit und ohne Partizipation dortiger Nutzer*innen entwickelt werden, riskieren, weniger passgenau die Bedarfe unterstützen zu können oder sogar nicht einsetzbar zu sein.

Ziel des vorliegenden systematischen Literaturreviews ist zu beschreiben, ob und wie das Thema partizipative Technologieentwicklung im Kontext Jugend- und Behindertenhilfe in, für die Soziale Arbeit relevanten, wissenschaftlichen Zeitschriften behandelt wird und so möglichst erschöpfend die Erkenntnisse entsprechender Forschungsprojekte zu eruieren. Zugleich zielt der Artikel darauf ab, die systematische Strategie der Literaturrecherche zu beschreiben.

## Methodik

Das vorliegende breite Review wurde zwischen Dezember 2019 und Mai 2020 durchgeführt. Die Recherche folgt dabei einem definierten Korpus und einem systematischen Vorgehen.

### Definition des Korpus

Zur Planung eines Forschungsprojekts ist es erforderlich, systematisch den Wissensstand zur beforschten Thematik – hier die partizipative Technologieentwicklung mit Jugendlichen in der Erziehungs- und Behindertenhilfe – zu erheben. Es ist zu erwarten, dass zu Erkenntnissen von Forschungsprojekten mindestens ein Artikel in einer einschlägigen wissenschaftlichen Zeitschrift publiziert wurde, sodass mit einer systematischen Zeitschriftenanalyse der State-of-the-Art umfassend bestimmbar sein sollte. Unter dieser Annahme ist es verzichtbar, unsystematisch Handbücher oder Monographien zu durchforsten. Zur Überprüfung dieses Vorgehens wurde eine Grobrecherche durchgeführt. Es zeigt sich, dass keine einschlägigen deutschsprachigen Handbücher oder Monographien zu partizipativer Technologieentwicklung im genannten Handlungsfeld auffindbar sind. Stattdessen muss methodisch begründet werden, in welchen Zeitschriften gesucht wird. Der fest definierte Korpus berücksichtigt die von der entsprechenden wissenschaftlichen Fachgesellschaft Deutsche Gesellschaft für Soziale Arbeit (DGSA) vorgeschlagenen Übersicht von 169 wissenschaftlichen Fachzeitschriften Sozialer Arbeit (Effinger et al. 2019) und die darin publizierte Artikel zwischen 1994 und Mai 2020. Die Eingrenzung auf die DGSA-Zeitschriftenliste begründet sich durch das Ziel der systematischen und nachvollziehbaren Literaturrecherche, die eine Beliebigkeit der Suche möglichst ausschließen soll.

Diese 169 Zeitschriften wurden zunächst anhand ihrer Themensetzung weiter selektiert und um jene Zeitschriften reduziert, die sich auf Thematiken fernab von Inklusion, Technik und Medien oder Behinderten- und Jugendhilfe beziehen. Resultierend daraus sind in 53 Zeitschriften thematische Überschneidungen vermutet worden, davon wurden schließlich 24 Zeitschriften der DSGA-Liste aufgrund der Stichwortsuche in die Recherche aufgenommen. Diese Selektion wurde um Zeitschriften jener Artikel ergänzt, die durch die breite Stichwortrecherche zusätzlich ermittelt wurden. Dabei erstreckte sich der Korpus auf insgesamt 31 Zeitschriften.

### Methode der Recherche

Für die Literaturrecherche ist im Vorhinein eine Strategie der Forschungsstandrecherche erstellt worden, welche eine Auswahl der relevanten Kernbegriffe enthält. Diese sind in Texten zu erwarten, die die Nutzung, die Weiterbildung, die Entwicklung von digitalen Medien und Technologien im Kontext einer auf Jugendliche bezogenen Behinderten- und Erziehungshilfe behandeln (Tab. 1). Jene wurden in zwei inhaltliche Schwerpunkte aufgeteilt, wodurch zum einen die Kategorie *Zielgruppe *(engl.: „target group“), zum anderen die zum *Objekt/Konzept (engl.: *„construct“*) *entstanden ist. Die deutschen Schlagwörter sind dabei mit einem englischen Äquivalent übersetzt worden, mit welchen englischsprachige Fachzeitschriften durchsucht worden sind.

**Table 1:** 

Zielgruppe	Objekt/Konzept	Target group	Construct
Beeinträchtig*	Bedürfnis*	Impair*	–
Behinder*	Digital Divide	Disabilit*/disabl*	Digital Divide
Benachteilig*	Bedarf*	Disadvantage*	Need*
Eingliederungshilfe	Digitale Ungleichheit	Integration assistance	Digital inequality
Erziehungshilf*	–	Educational Support	–
Handicap*	Assistenzsystem	Handicap*	Assist*e System
Heimerzieh*	Assistenztechnologi*	Foster care	Assist*e technolog*
Hilfe* zur Erziehung	Quantified Self	Educational support	Quantified Self
Jugendhilf*	Inklusi* Techn*entwicklung	Youth welfare*	Inclusi* technolog* development
Jugendliche*	Partizip* Techn*entwicklung	Youth	Participat* technolog* development
Junge* Erwachsene*	Unterstützungstechnologi*	Young adult*	Supportive technolog*
Kinder- und Jugendhilfe	Digitalisier*	Child and youth welfare	Digita*ation
Soziale Einrichtung	Digitale Teilhabe	Social facilities	Digital participation
Stationär* teilstationär*	Medienpädagog*	(Semi-) residential care	Media pedagog*
Wohneinrichtung	–	Residential facilit*	–
Wohngruppe	Digitale Kluft	Residential group*s	Digital gap
–	Smart Home	–	Smart Home

Zunächst fand eine grobe Durchsuchung der Zeitschrift statt, indem breite Überbegriffe (ODER-Verknüpfungen wie z. B. Tech*, Medien*, Digi* …) gebildet worden sind. So wurde herausgefunden, ob in der vorliegenden Zeitschrift überhaupt Artikel mit Nähe zur Forschungsthematik vorliegen. Wenn thematische Treffer gefunden worden sind, wurde die Zeitschrift einer intensiveren Durchsicht mit spezifischen Schlagwortkombinationen (UND-Verknüpfungen; Tab. 1) unterzogen. Um Forschung mit Verknüpfung der beiden Schwerpunktkategorien *Zielgruppe *und *Objekt/Konzept* zu finden und somit die potenziell relevanten Artikel für das Thema partizipative Technikentwicklung mit Jugendlichen auszufiltern, wurden in den Suchvorgängen jeweils zwei Suchbegriffe – je einer pro Kategorie – miteinander kombiniert (UND-Verknüpfung).

## Ergebnisse

Es wurden thematische und quantitative Ergebnisse vorgefundener Zeitschriftenartikel festgehalten, die im Folgenden erläutert und dann diskutiert werden. Eine tabellarische Darstellung der Ergebnisse findet sich auf INTIA (2022). Die Tabellen auf der Webseite stellen die Artikel kategorisiert sowie nach durchsuchten Zeitschriften dar.

### Qualitative Ergebnisdarstellung

Die systematische Analyse der identifizierten Artikel zeigt unterschiedliche thematische Forschungslinien auf, die in vier inhaltliche Themenstränge kategorisiert wurden. So ist die Kategorie 1 „partizipative Forschung“ in folgende Subkategorien „partizipative Jugendforschung“, „partizipative Forschung mit Kindern und Jugendlichen mit Behinderung“ und „grundlegende (ethische) Vorgehensweisen partizipativer Forschung“ untergliedert. Die Überkategorie 2 „Assistenztechnologien“ besteht aus den Subkategorien „technologische Lernunterstützung“, „Assistenztechnologien für Menschen mit Behinderung“ „für ältere Menschen“ und „allgemein Theorie und Empirie zu Assistenztechnologien“. Die übergeordnete Kategorie 3 „Handlungsanweisungen für Medienkompetenz von Jugendlichen“ ist untergliedert in „Handlungsweisen für Medienkompetenz“, „Mediennutzung“, „schulische Integration durch Technologien“ und „mediatisierte Lebenswelten“. Mit der Forschungslinie „Digitalisierung in der Sozialen Arbeit“ wurde eine weitere Kategorie 4 identifiziert, die sich mit „dem Verhältnis von Technologien und Sozialer Arbeit“ auseinandersetzt sowie dem „digital divide“.

In der Kategorie 1 *partizipative Forschung* finden sich Artikel zur *partizipativen Forschung mit Kindern und Jugendlichen mit Behinderung*, zur *partizipativen Jugendforschung* und zu *grundlegenden (ethischen) Vorgehensweisen* der partizipativen Forschung. Lediglich vier Publikationen thematisieren dabei partizipative Technologienentwicklungen: Zwei der Artikel beziehen sich dabei auf Entwicklungsprozesse mit Jugendlichen, die eine App entwickeln und im Anschluss evaluieren. Durch die partizipativen Projekte lernen die Jugendlichen nicht nur Prozesse einer App-Entwicklung technisch kennen, sie reflektieren zudem die Nutzung von Messengerdiensten (Rosenbaum et al. 2017). Damit findet durch die Partizipation ein informierter Umgang mit den Technologien statt und das Potenzial einer selbstbestimmten reflektieren Lebensführung wird deutlich. Es zeigt sich durch die Partizipation der Zielgruppen, dass sich die Anliegen von Forschenden und Nutzenden stark unterscheiden können: Bei einer gemeinsamen Entwicklung von Skalen für eine Smartphone-App zeigt sich eine Diskrepanz der Vorstellungen. Während die Sozialarbeiter*innen die Stärken und Potenziale der Jugendliche hervorheben möchten, ist es der Wunsch der Jugendlichen, die alltäglichen Herausforderungen ihrer Lebensführung abzubilden (Mackrill et al. 2015). Dieser Punkt weist daraufhin, stets die Perspektiven der Nutzenden miteinzubeziehen, sodass die entwickelten Technologien möglichst passgenau auf die Bedürfnisse dieser abgestimmt sind. Allsop et al. (2011) bedienen sich webbasierter Umfragemethoden zur Entscheidungsteilhabe bei Hilfsmitteln in der Grundschule. In allen drei Artikeln finden Jugendliche in stationären Wohnformen keine Berücksichtigung. Die vierte Veröffentlichung zur Partizipation in Technologieentwicklungen mit vulnerablen Personengruppen beschreibt das User Involvement von behinderten Menschen in Produkttestungen. Die Annahmen der Studien können weder bestätigt noch widerlegt werden, sodass die Autor*innen als Ergebnis die Empfehlung formulieren, weiterführende Forschungen zur partizipativen Technikentwicklung zu unternehmen (Borch und Strandbakken 2019).

Der Bedarf an Forschung bestätigt sich ebenfalls durch Artikel der Kategorie 2 *Assistenztechnologien*: In Bezug auf innovative Prozesse in der Implementierung dieser Technologien wird die Notwendigkeit auf anschließende Forschung deutlich (Taherian und Davies 2017; McDowell 2015; Passey 2015). Die Publikationen vereinen die dringliche Empfehlung, weiter an der Technikentwicklung zu forschen. Der verstärkte Einsatz von Assistenztechnologien wird dabei als eine Teilhabestrategie in der Literatur diskutiert. Smith et al. (2018) beschreiben die Grundvoraussetzungen der Implementierung, Winberg et al. (2019) und Borg et al. (2011) formulieren ethische Aspekte, Ripat et al. (2020), Gjessing et al. (2018) und da Silva et al. (2018) plädieren für die Notwendigkeit der Erschwinglichkeit von Assistenztechnologien. Parsons (2015) stellt eine neu gewonnene Autonomie der Nutzenden durch die Unterstützung der Selbstständigkeit durch assistive Technologien heraus. Dies schlussfolgern auch Brandt et al. (2011), die den Einsatz von Technologien in Verbindung mit einer steigenden Lebensqualität setzen. Auch wenn der Grad der Evidenz als niedrig eingeschätzt wurde, geben die Untersuchungen vielversprechende Hinweise auf eine höhere Selbstständigkeit, häufigere Teilnahme an Freizeitaktivitäten und somit eine gesteigerte Lebensqualität der Studienteilnehmenden insbesondere durch Technologien für *Smart Homes* und Umgebungssteuerung. Die Autor*innen liefern damit Argumente zur Relevanz von digitalen Assistenzsystemen, welche für die partizipative Technologieentwicklung in der Eingliederungshilfe relevant sein könnten. Pedersen et al. (2019) verdeutlichen mit ihren Untersuchungen, dass digitale Unterstützungssysteme die Teilhabe im Alltäglichen erleichtern können. Die Systeme ermöglichten dabei neue Tagesabläufe und damit Routinen, die wiederum neue (zeitliche) Ressourcen freistellen. Die gewonnene Zeit sowie die Unterstützungsmöglichkeiten nutzten die Betroffenen individuell und selbstbestimmt für persönliche Bedürfnisse.

Die Kategorie 3 *Handlungsanweisungen für Medienkompetenz* veranschaulicht die Priorisierung von Medienkompetenz in Jugendhilfe: Eine Vielzahl der Artikel thematisieren die mediale Aus- bzw. Weiterbildung von Jugendlichen sowie Fachkräften der Sozialen Arbeit – darunter beziehen sich einige Artikel auf jugendbezogene Behinderten- und Erziehungshilfen. Die Artikel stellen die Notwendigkeit eines Umdenkens in den stationären Wohnformen heraus: Statt Restriktionen und Sanktionen müssen den Jugendlichen Kompetenzen vermittelt werden, um reflektiert digitale Technologien nutzen zu können. Die Autor*innen formulieren einen Handlungsbedarf zur aktiven Begleitung der Jugendlichen im Rahmen digitaler Prozesse (Erhard 2018; Tillmann 2018; Brokmeier 2015; Hajok 2015). Die Partizipation an Technologieentwicklungen (im Vergleich zur Mediennutzung) wird dabei nicht berücksichtigt. Die partizipative Teilnahme an solchen technologischen Prozessen lässt aber bedeutsamen Kompetenzerwerb vermuten. Die Artikel zu mediatisierten Lebenswelten verdeutlichen den kulturellen Wandel in der Medienumgebung von Kindern und Jugendlichen. Digitale Teilhabe ist relevant für unterschiedliche Teilhabechancen im gesellschaftlichen Miteinander (Tillmann 2017; Ebel 2017). Digitale Medien verändern die Lebenswelt von Klient*innen (in Einrichtungen) der Sozialen Arbeit: Kutscher (2018) diskutiert einen (schleichenden) Gesellschaftswandel in Form der Digitalisierung und beschreibt dabei relevante Handlungsfelder für die Entwicklung der Sozialen Arbeit auf. Darunter zählen Prägung der Lebenswelten durch Soziale Medien; Verwendung digitalisierter Informationsverarbeitung; Onlineberatung sowie softwarebasierte Diagnostik und Risikoeinschätzungsverfahren. Sie schlussfolgert, das Forschungsfeld der Digitalisierung im Kontext der Sozialen Arbeit sei näher zu beleuchten.

Die *Digitalisierung in der Sozialen Arbeit* als Kategorie 4 zeigt anhand unterschiedlicher Artikel auf, welche Bedeutung die Digitalisierung für die Soziale Arbeit haben kann. Diese Kategorie ist inhaltlich kaum von den mediatisierten Lebenswelten zu unterscheiden. Geyer (2018) beschreibt die Digitalisierung im Kontext von Teilhabechancen für Klient*innen der Sozialen Arbeit. Er skizziert neue Möglichkeiten für die Soziale Arbeit und macht zugleich darauf aufmerksam, dass diese eine digitale Theorie und Praxis benötigt. Das notwendige Anpassen von Wahrnehmung und Denken schließt auch Fachkräfte nicht aus. Da in sozialen Hilfeeinrichtungen die Haltung der dort arbeitenden Sozialarbeiter*innen ausschlaggebend für die Akzeptanz und Implementierung digitaler (Assistenz‑)Technologien sind, thematisieren Goldkind et al. (2016) in ihrer Publikation die Einstellung der Fachkräfte sowie das Umsetzen von Nutzungskonzepten. Dabei stoßen die Autor*innen auf individuellen Widerstand gegen Innovation bzw. Veränderung. Zugleich zeigen die Fachkräfte Lücken in der Infrastruktur auf und kommen damit zu einer Erkenntnis, die ebenso in anderen Zeitschriftenartikel herausgearbeitet wurde: Die Ausstattung in (jugendbezogenen) stationären Hilfseinrichtungen ist unzureichend. Digital Divide als digitale Ungleichheit und Spaltung wird im Kontext von (jugendbezogener) Sozialen Arbeit diskutiert (Gögercin 2001). Eingeschränkte Zugangsmöglichkeiten zu digitalen Technologien und unterschiedliche Befähigung zur Nutzung dieser können Risiken für soziale Exklusion mit sich bringen (Steyaert und Gould 2009). Dies ist umso mehr für ohnehin schon benachteiligte Personengruppen zu befürchten. Dabei zeigen einige Artikel explizit die sozialen Ausgrenzungsmuster für behinderte Menschen in der Digitalisierung auf (Henne 2019; Reichstein 2016; Macdonald und Clayton 2013). Das Exklusionsrisiko wird jedoch in den selektierten Artikeln nicht im Rahmen von jugendbezogenen Eingliederungs- und Erziehungshilfen diskutiert.

### Quantitative Ergebnisdarstellung

Quantitativ zeigt sich folgendes Bild entlang der Ergebniskategorien: Insgesamt wurden 158 im weitesten Sinne relevante Artikel in den aufgelisteten 31 Zeitschriften ermittelt, wobei sich die Recherche im Gesamten auf 53 Zeitschriften erstreckte (Details siehe INTIA 2022). Die kategoriale Darstellung der Artikel zeigt, zu welchen Kategorien sowie Unterkategorien mehr oder eher weniger viel geforscht wird:

Zur Forschungslinie der *partizipativen Forschung* wurden 27 Artikel gefunden, davon 14 in der Subkategorie *partizipativen Forschung mit Menschen/Kindern mit Beeinträchtigung, *sieben zu *partizipativer Jugendforschung *und sechs Beiträge stellen *grundlegende (ethische) Vorgehensweisen partizipativer Forschung* vor. Lediglich vier dieser Publikationen beziehen sich in der partizipativen Forschung auf *partizipative Technik‑/Medienentwicklung *(Borch und Strandbakken 2019; Rosenbaum et al. 2017; Mackrill et al. 2015; Allsop et al. 2011).

Die zweite übergeordnete Kategorie *Assistenztechnologien* umfasst 55 relevante Artikel, wovon die Subkategorie *technologische Lernunterstützung* mit 18 Beiträgen einen hohen Bestandteil einnimmt. Zu *Assistenztechnologien* speziell für *Menschen mit einer Behinderung bzw. vulnerablen Gruppen* wurden 16 Veröffentlichungen gefunden. Außerdem konnten zu *digitalen Technologien für Ältere* elf Artikel ermittelt werden, wobei der Fokus auf der Auseinandersetzung mit der Demenzthematik liegt. Eine weitere Subkategorie zu *Theorie und Empirie in Assistenztechnologien *zählt elf Artikel.

Die Kategorie *Handlungsanweisungen für Medienkompetenz* schließt 23 Artikel ein. Dabei gliedert sie sich in die Subkategorien *Handlungsweisen für Medienkompetenz* und *Mediennutzung*. Die Forschung zu Medien in (jugendbezogenen) Eingliederungs- und Erziehungshilfen ist stark von Handlungsweisen für Medienkompetenz von Jugendlichen geprägt. Die Subkategorie *Handlungsweisen für Medienkompetenz* umfasst zwölf Artikel, während die *Mediennutzung* nur sieben Artikel zählt. Explizit zur (stationären) Erziehungshilfe konnten vier Beiträge identifiziert werden (Erhard 2018; Tillmann 2018; Brokmeier 2015; Hajok 2015) – zur Medienkompetenz in der Behindertenhilfe hingegen nur eine Veröffentlichung (Bosse et al. 2019). Zu den *mediatisierten Lebenswelten* wurden fünf Artikel gefunden.

In der Überkategorie zur *Digitalisierung in der Sozialen Arbeit* wurden 18 Publikationen gefunden, wovon zehn Beiträge das *Verhältnis digitaler Technologien und Sozialer Arbeit* herausarbeiten. Zu Themen der Subkategorie *Digital Divide* im Kontext der Sozialen Arbeit und *digitalen Teilhabe von benachteiligten Zielgruppen* sind in den durchsuchten Zeitschriften acht Beiträge gefunden worden.

### Diskussion der Ergebnisse

Eine Handlungsempfehlung zur weiterführenden Forschung bezüglich der partizipativen Technikentwicklung in jugendbezogenen Behinderten- und Erziehungseinrichtungen wird durch die kategoriale Darstellung und Diskussion der Artikel sichtbar: Es finden sich Artikel zum partizipativen Forschen mit vulnerablen Menschen (Borch und Strandbakken 2019; Rix et al. 2019; Tran und Brodersen 2019; Daniel 2018), jedoch beziehen sich lediglich Borch und Strandbakken (2019) auf partizipative Technikentwicklung. Assistive Technologien werden bislang vor allem im schulischen Kontext, aber weniger im stationären Setting erforscht (Nordström et al. 2019; Nepo 2017; McDowell 2015; Passey 2015). Der Diskurs zu digitalen Unterstützungssystemen in der stationären Erziehungshilfe ist wenig ausgeprägt. Dies ist umso erstaunlicher, als digitale Unterstützungssysteme von der Breite der Bevölkerung genutzt werden, z. B. Navigationshilfen, Finanzplaner, Erinnerungsmanagement, Termin- und Einkaufsplaner, Smart-Home-Technologien. Der Bereich der Behindertenhilfe wird zwar abgedeckt (Jamwal et al. 2020; Levasseur et al. 2016), diese Projekte finden jedoch häufig in außerstationären Settings statt (Munde 2017; Walters et al. 2015). Digitale Assistenzsysteme sind stärker in der Pflege – insbesondere bei Demenzerkrankungen – erforscht (Tsertsidis 2020; Fehling 2019; Lenz et al. 2019; Weber 2017; Beer et al. 2015; Landau et al. 2009). Die Recherche bildet ab, dass vor allem schulische Settings und pflegerische Bereiche partizipative Forschungskontexte anwenden – mit Themenschwerpunkten jenseits der Technik- oder Medienentwicklung. Obwohl partizipative Technologieentwicklung insbesondere für die diskutierten Handlungsfelder in der stationären Jugend- und Eingliederungshilfe weitreichende Potenziale verspricht, findet dieser Bereich in der Forschung kaum Berücksichtigung. Die Kategorie *Digitalisierung in der Sozialen Arbeit *stellt dabei fest, dass in stationären Einrichtungen Ausstattungs- und Nutzungskonzepte mangelhaft sind. Dadurch wird der Ausschluss von sozialer sowie digitaler Teilhabe der Jugendlichen riskiert (Steyaert und Gould 2009).

Um die Schlussfolgerung zu validieren und Ergebnisse des Literaturreviews ergänzen zu können, werden neben dem ausgewählten Zeitschriftenkorpus der Recherche einschlägige Werke zum Forschungsfeld hinzugezogen, darunter das umfassende Handbuch zu Digitalisierung und Soziale Arbeit (Kutscher et al. 2020). Bei der Untersuchung von Forschungsperspektiven und dem Stand des empirisch gestützten Wissens zu digitalen Medien in stationären und jugendbezogenen Hilfeeinrichtungen zeigen Tillmann und Weßel (2021) ein Forschungsdesiderat in Bezug auf Handlungsweisen und Interaktionen mit digitalen Medien von Jugendlichen, Eltern und Fachkräften auf, die unterschiedliche und teils konfligierende Ziele verfolgen. Wenn Forschungsperspektiven untersucht werden, dann fokussieren sich die Forschungen zumeist auf einzelne Akteur*innen. Einige wenige Studien zeigen Dynamiken zwischen Jugendlichen und Fachkräfte auf. Im deutschsprachigen Raum ist die digitale Medienbildung von Jugendlichen in stationären Wohnformen unterrepräsentiert. Kutscher und Siller (2020) führen weiter aus, dass Digitalisierungsprozesse in Abhängigkeit vom Forschungsfeld in der Sozialen Arbeit optimistisch (z. B. Jugendarbeit) oder skeptisch (z. B. Kindertagesbetreuung) betrachtet werden. Skepsis gegenüber Technologieentwicklungen zeigen sich insbesondere in Veröffentlichungen zu computergestützten Verfahren der Risikodiagnostik bezüglich des Kindesschutz (Schrödter et al. 2020; Ackermann 2020). In den (hier im Artikel auszugsweise schon genannten) Publikationen der International Conference for Computing Helping People with Special Needs (ICCHP) findet sich eine Vielzahl aussagekräftiger und relevanter Projektberichte zur Thematik (beispielsweise Miesenberger et al. 2022), die bislang nicht in Zeitschriften der Sozialen Arbeit rezipierbar und diskutiert sind. Als relevante Erkenntnis ist festzuhalten, dass keine einschlägigen deutschsprachigen Handbücher oder Monographien zu partizipativer Technologieentwicklung mit Jugendlichen in stationären Einrichtungen der Sozialen Arbeit auffindbar sind.

## Fazit

Ziel der Studie war es herauszufinden, wie die Thematik partizipativer Technologieentwicklung in Einrichtungen der Jugend- und Behindertenhilfe aus einer Perspektive der Sozialen Arbeit behandelt wird. Als beforschte Felder und Themen fanden wir: Partizipative Forschung, Assistenztechnologien für behinderte und für ältere Menschen und Theorien zu Assistenztechnologie, Handlungsanweisungen für Medienkompetenz von Jugendlichen und von Fachkräften, Perspektiven auf mediatisierte Lebenswelten sowie auf die Problematiken eines diesbezüglichen Digital Divide. Mit der Forschungslinie Digitalisierung in der Sozialen Arbeit wurde eine weitere Kategorie identifiziert, die sich mit dem Verhältnis von Technologien und Sozialer Arbeit auseinandersetzt. Methoden partizipativer und/oder inklusiver Technikentwicklung für Kontexte der Sozialen Arbeit sind bislang kaum erforscht. Allerdings sind partizipativ entwickelte Methoden und Technologien in Kontexten von Pflege, Schule oder privater Raum bereits implementiert und können Erfolge in der Inklusion ihrer Nutzer*innen erzielen. Die Relevanz und das Potenzial für Soziale Arbeit müssen diskutiert werden, um technische Innovationen bei der Hilfeerbringung eruieren und die Passgenauigkeit der Entwicklungen durch die Partizipation der Nutzenden ermöglichen zu können. Nicht zuletzt die durch die COVID-19-Pandemie sichtbar gewordenen Teilhabeproblematiken stationärer Einrichtungen bestärkt die Notwendigkeit der Erforschung partizipativer inklusiver Technikentwicklungsprozesse in der Sozialen Arbeit. Mit den Zielen, einerseits digitale Inklusion gezielt Kindern und Jugendlichen aus der Behinderten- und Erziehungshilfe zu ermöglichen sowie andererseits die Digitalisierung in Einrichtungen inklusiv und partizipativ zu gestalten, sollten Erkenntnisse von bereits bestehenden Forschungen, wie Pflege und Schule, geprüft werden, bezüglich ihrer Anwendbarkeit auf das spezifische Handlungsfeld der jugendbezogenen Hilfseinrichtungen im Kontext der Sozialen Arbeit. Diese Forschungserkenntnisse zeigen verschiedene Potenziale der partizipativen Technologieentwicklung wie der Gewinn neuer zeitlicher Ressourcen, Autonomie oder das Erreichen einer selbstständigen Lebensführung auf. Zudem finden sich relevante und weiterführende Forschungen in Publikationen aus Informatik, Design und der Konferenz International Conference on Digital Inclusion, Assistive Technology (ICCHP). Es ist daher anzuregen, dass Verknüpfungen dieser disziplinären Kenntnisbestände mit jenen der Sozialen Arbeit hergestellt werden, da ansonsten riskiert wird, dass der Sozialen Arbeit Potenziale für ihre bestmögliche Aufgabenerfüllung verborgen bleiben.
